# Evolutive Study of Dietary Aspects and Intestinal Microbiota of Pediatric Cohort with Cow’s Milk Protein Allergy

**DOI:** 10.3390/children11091113

**Published:** 2024-09-12

**Authors:** Ana M. Castro, Sandra Navarro, Ignacio Carvajal, Agueda García, Marta Suárez, Paula Toyos, Silvia Rodríguez, Santiago Jimenez, David González, Cristina Molinos, David Pérez-Solís, Porifirio Fernández, Abelardo Margolles, Juan J. Díaz-Martín, Isabel Gutiérrez-Díaz, Susana Delgado

**Affiliations:** 1 Grupo MicroHealth, Instituto de Productos Lácteos de Asturias (IPLA-CSIC), 33300 Villaviciosa, Asturias, Spain; ana.castro@ipla.csic.es (A.M.C.); amargolles@ipla.csic.es (A.M.); isabel.gutierrez@ipla.csic.es (I.G.-D.); sdelgado@ipla.csic.es (S.D.); 2 Instituto de Investigación Sanitaria del Principado de Asturias (ISPA), 33011 Oviedo, Asturias, Spain; 3 Pediatría, CAP Teatinos-Corredoria, 33011 Oviedo, Asturias, Spain; sandra.navarro@sespa.es; 4 Pediatría, CAP La Eria, 33013 Oviedo, Asturias, Spain; ignacio.carvajal@sespa.es; 5 Pediatría, CAP Vallobin-La Florida, 33012 Oviedo, Asturias, Spain; agmerino@telefonica.net; 6 Pediatría, Hospital Universitario Central de Asturias (HUCA), 33011 Oviedo, Asturias, Spain; marta.suarezggo@sespa.es (M.S.); paula.toyos@sespa.es (P.T.); david.gonzalezj@sespa.es (D.G.);; 7 Pediatría, Hospital Universitario de San Agustín, 33401 Avilés, Asturias, Spain; silvia.rodriguez@sespa.es (S.R.); david@perezsolis.es (D.P.-S.); 8 Pediatría, Hospital Universitario de Cabueñes, 33394 Gijón, Asturias, Spain; cristina.molinos@sespa.es

**Keywords:** CMPA, food allergy, intestinal microbiota, immune system, diet

## Abstract

Background: One of the most common food allergies in the pediatric population is allergy to cow’s milk protein (CMPA). Treatment consists of avoiding cow’s milk proteins in lactating mothers and/or using therapeutic formulas based on hydrolysates or vegetable formulas. In infants with CMPA at diagnosis, a different gut microbial profile has been found compared to healthy children, with a reduction in beneficial bacteria. The aim of this study was to evaluate changes in the gut microbiota profile and its metabolites, dietary patterns and anthropometric variables in a pediatric cohort with CMPA after six months on a restrictive diet compared to healthy controls. Methods: In total, 21 patients diagnosed with CMPA and a control group of 24 healthy infants participated in this study. The fecal microbiota of all participants were investigated by metataxonomic analysis of 16S rDNA amplicons, and fecal short-chain fatty acids were measured by gas chromatography. Epidemiological assessment and dietary questionnaires were carried out for both groups. Results: Regarding growth, no significant differences were found, but differences in dietary intake of some macro- and micronutrients were observed. Patients who were breastfed at six months had higher bifidobacteria and lipid intakes than patients fed with hydrolyzed formulas. Conclusions: Although the growth of CMPA infants fed with therapeutic formula is similar to breastfed CMPA infants, there are differences in microbiota composition and macronutrient intake that underline the importance of continued breastfeeding in CMPA cases.

## 1. Introduction

Cow’s milk protein allergy (CMPA) represents a prevalent immune-mediated adverse reaction to proteins present in cow’s milk. This allergy occurs when the immune system identifies specific proteins in cow’s milk, such as casein and whey proteins (β-lactoglobulin and α-lactoalbumin), as harmful invaders, thereby triggering an immune response [1,2]. The condition is most prevalent in infants and young children, but it can persist into adulthood. In children, the prevalence of the allergy is estimated to be between 3 and 8%, but this tends to decrease with age, with an estimated prevalence of 0.16% and 0.49% in older children and adults, respectively [3,4,5].

The symptoms of CMPA can vary widely, ranging from mild to severe reactions [6,7]. These may include skin rashes, hives, digestive issues such as vomiting or diarrhea, respiratory problems such as wheezing or nasal congestion and, in severe cases, anaphylaxis. The fundamental tenets of CMPA treatment entail the exclusion of cow’s milk protein (CMP) from the infant’s diet. This can be achieved through a variety of approaches in both breastfed and formula-fed infants [8,9]. It is recommended that breastfeeding be maintained while dairy products are excluded from the mother’s diet, and that the mother receives adequate calcium and vitamin D supplementation. In cases where breastfeeding is not possible, a hypoallergenic formula should be used. As a first line of treatment, the recommended formulas are extensively hydrolyzed casein or whey and hydrolyzed rice formulas. Soy formulas could also be considered a second-line option. In severe cases or when first-line formulas fail, amino acid-based formulas should be used [10,11].

The first three years of life represent a critical period for the development of the gut microbiota. This is influenced by a number of factors, including the mode of birth, the use of antibiotics and/or the type of feeding. In this context, breastfeeding is regarded as the gold standard for infant nutrition in early life. The presence of human milk oligosaccharides (HMOs) in breast milk has been shown to facilitate the growth of beneficial bacteria, while simultaneously inhibiting the adherence of pathogens to the intestinal epithelium [12]. Several studies have shown that the composition and diversity of the gut microbiota may influence susceptibility to food allergies [13]. Infants with CMPA have been observed to have differences in the composition of their gut microbiota compared to healthy children [14,15]. These differences include reduced microbial diversity, alterations in specific bacterial taxa (such as reduced abundance of bifidobacteria) and variations in the production of microbial metabolites. Nevertheless, the precise manner in which the microbiota influences the development of CMPA and vice versa remains a topic of ongoing investigation and is not yet fully established.

Therefore, the aim of this study was to assess the impact of a six-month dietary intervention on changes in the gut microbiota profiles and their metabolites, dietary patterns and anthropometric variables in a pediatric population with CMPA, compared to healthy controls (HCs).

## 2. Materials and Methods

### 2.1. Study Design and Participants

The general design of the trial has been previously published [15]. In summary, this study was conducted with a pediatric cohort diagnosed with CMPA. The cohort was monitored for six months following diagnosis and treated with a restricted diet during this period. Of the 27 patients originally recruited, 6 were lost to follow-up. The study population comprised a group of 21 pediatric patients with CMPA (P-CMPA) followed up with in three reference hospitals in northern Spain (Principality of Asturias, Spain) and 24 HCs recruited and followed up with during routine wellness childcare visits in primary care centers. Inclusion criteria for patient selection were as previously published [15] and included hospital diagnosis according to the guidelines of the European Society for Paediatric Gastroenterology, Hepatology and Nutrition (ESPGHAN) [11]. Exclusion criteria included the concomitant presence of other food allergies, enteropathies or any other cause of diarrhea at the time of inclusion, as well as the use of antibiotics or corticosteroids in the month prior to stool collection were considered. The parents or legal guardians of all participants were informed of the details of the study and gave their written consent to take part. This study was approved by the Regional Ethics Committee for Clinical Research of Asturias (CEI-PA) (Ref. 343/19). The clinical data of the participants were collected using Research Electronic Data Capture (REDCap) tools [16], hosted at “Sociedad Española de Gastroenterología, Hepatología y Nutrición Pediátrica” (SEGHNP) (https://redcap.seghnp.org/, accessed on 1 July 2024) with assistance from the AEGREDCap Support Unit, and shared with “Asociación Española de Gastroenterología” (AEG).

### 2.2. Nutritional and Anthropometric Assessment

A three-day food record was used to collect the infant’s dietary information. This included all meals consumed by the infant over a 3-day period, including a weekend day. A registered dietitian recorded comprehensive data regarding the initial food items and the month in which they were introduced to the infant diet. Moreover, supplementary data pertinent to the study, such as fruit consumption with or without the peel, were gathered. Energy content and nutrient composition were calculated using a Spanish dietary analysis website, “Organizador Dietético Metabólico (ODIMET)” [17]. After diagnosis, patients were given recommendations for the dietary management of CMPA. While some patients continued to breastfeed, others were recommended to use special therapeutic formulas. In this case, the feeding types used in the study were classified as follows: breastfeeding (BF), extensively hydrolyzed milk formulas (EHF) including amino acid-based formulas, and hydrolyzed rice formulas (RF). Children’s height (cm) and weight (kg) were recorded to the nearest 0.1 cm and 0.1 kg, respectively, by a pediatric nurse using calibrated and approved equipment at all time points analyzed. Body mass index was calculated as kg/m^2^. Anthropometric measurements, including weight-for-age (WFA), height-for-age (HFA) and body mass index-for-age (BMI) were obtained according to the WHO Child Growth Standards, adjusted for gender and age, using WHO ANTHRO Software for Calculating Anthropometry, version 3 [18,19] (Z-score = average value ± SD). The normal upper and lower limits were defined as values between +2SD and −2SD, respectively [20].

### 2.3. Fecal DNA Extraction and Quantification

Stool samples were homogenized in phosphate-buffered saline (PBS) to obtain a 1:10 dilution used for DNA extraction. This method was performed according to the International Human Microbiome Standards (IHMS), protocol Q [21], using the QIAamp DNA Stool minikit (Qiagen, Hilden, Germany) with minor modifications, as previously reported [15]. These modifications mainly concern the lysis steps, were performed using a Fastprep FP24 homogenizer (Biomedicals, Irvine, CA, USA), and consisted of 3 cycles of 45 s each, leaving the samples on ice for 5 min between each cycle. Finally, the eluted DNA (100 µL) was measured using a Qubit dsDNA BR assay kit (Thermo Fisher, Waltham, MA, USA).

### 2.4. High-Throughput Sequencing of 16S rRNA Gene Amplicons

The sequencing of 16S rRNA gene amplicons was performed from the fecal DNA samples with primers pair “Probio_Uni/Probio_Rev” targeting the V3 region of the 16S rRNA gene according to Milani et al. using an Illumina MiSeq System (Illumina, San Diego, CA, USA) at the spin-off of the University of Parma Genprobio srl (Italy) [22]. The quality-approved, trimmed and filtered sequences were processed and classified using the Quantitative Insights Into Microbial Ecology (QIIME) software suite v.2 and were classified to the lowest possible taxonomic rank considered, using SILVA database v. 132 as a reference.

### 2.5. Fecal Short-Chain Fatty Acids (SCFAs) Determination

A 1:2 dilution of the feces in PBS was used for the determination of SCFAs. This dilution (100 µL) was combined with 50 μL of 2-ethyl butyric acid (Sigma-Aldrich, San Louis, MI, USA), which was used as the internal standard (1.05 mg/mL in methanol), and acidified with an additional 50 μL of 20% formic acid (*v*/*v*). The acidic solution was then extracted with 450 µL of methanol and centrifuged for 10 min at 16,300× *g*. The supernatants were stored at −20 °C until analysis by gas chromatography (GC). The system and conditions were those previously described [23]. The chromatographic system consisted of a 6890 GC injection module (Agilent Technologies, Santa Clara, CA, USA) with a HP-FFAP (30 m × 0.250 mm × 0.25 μm) column (Agilent Technologies), and detection was performed with a flame ionization detector (FID). Data acquisition and processing were performed using ChemStation Agilent G1701DA software (Agilent Technologies).

### 2.6. Statistical Analyses

Statistical analyses were performed using IBM SPSS Statistics v.28.0.1 (IBM, Armonk, NY, USA) and RStudio software version 4.3.0 (R Foundation for Statistical Computing, Vienna, Austria). The Kolmogorov–Smirnov test was used to assess the suitability of the data for normal distribution. Categorical variables were expressed as proportions, and the chi-squared test was used for comparisons between groups. For continuous variables, expressed as means and standard variances, the Mann–Whitney U and Kruskal–Wallis tests were used to evaluate differences. Paired *t*-tests or Wilcoxon’s signed rank tests were used for comparisons over time. Two-sided probability values of *p* ≤ 0.05 were considered significant. Origin Pro was used to create evolutionary graphics.

## 3. Results

### 3.1. Participant Characteristics

**Figure 1:**
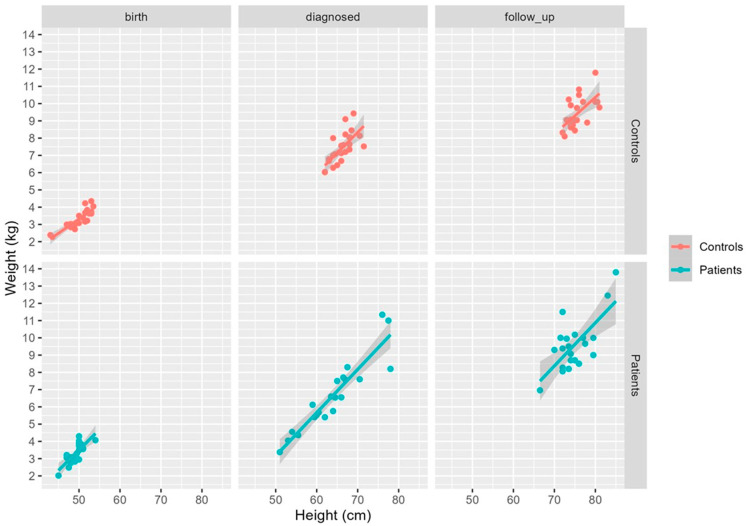
Growth performance in terms of weight and height at birth, diagnosis and 6 months’ follow-up for controls and patients.

For the analysis of the results, the data were processed according to P-CMPA and HC. Information on gender, age, anthropometric measurements, and other variables of interest for both groups are shown in Table 1. The data showed no significant differences according to gender, age, or type of delivery between patients and controls. Regarding allergy type, 57.10% of patients had IgE-mediated CMPA, whereas 42.90% had non-IgE-mediated CMPA (Table 1). More than half of the patients used an extensively hydrolyzed milk formula (EHF), 19.00% of patients used rice formula (RF), while another 19% of children with CMPA continued to breastfeed (BF) (Table 1). In the control group, the infants were fed either breast milk or regular follow-on infant formula, which were included in the “others” category (Table 1). Regarding growth, no significant differences were observed in the anthropometric variables at birth, diagnosis and follow-up, indicating a comparable growth pattern in patients and controls (Figure 1).

### 3.2. Nutritional Assessment

First, a general descriptive study was conducted to compare the dietary patterns of the controls and the patients. The dietary assessment revealed discrepancies in dietary intakes of specific macronutrients between the two groups (Table 2). A reduction in lipid and saturated fatty acid (SFA) consumption was observed in the P-CMPA subjects compared with the dietary habits of the HC. With regard to micronutrients, the intake of specific vitamins, including vitamin C, vitamin E, vitamin B3 and vitamin K, was found to be lower in the HCs than in the P-CMPA group (Table 3).

**Figure 2:**
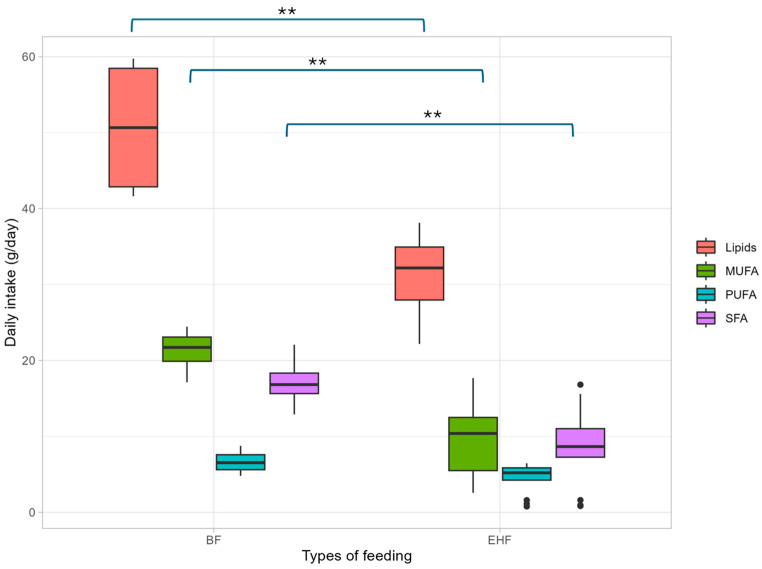
Profile of the lipid intake according to the type of feeding of the patients. SFAs, saturated fatty acids. MUFAs, monounsaturated fatty acids. PUFAs, polyunsaturated fatty acids. Asterisks ** indicate significant statistical differences (*p* value ≤ 0.01).

Given the diversity of feeding practices among patients, three nutritional profiles were created to align with the specific feeding types utilized in this pediatric population. Differences in the intakes of lipids, SFAs and monounsaturated fatty acids (MUFA) between BF and EHF-fed patients were observed, with the latter having lower intakes of these dietary components (Figure 2). Regarding micronutrients, vitamin C intake was higher in EHF-fed patients compared to breastfed patients (*p* value = 0.017). However, no significant differences were observed for the other dietary components.

### 3.3. Fecal Microbiota and Compositional Profile

**Figure 3:**
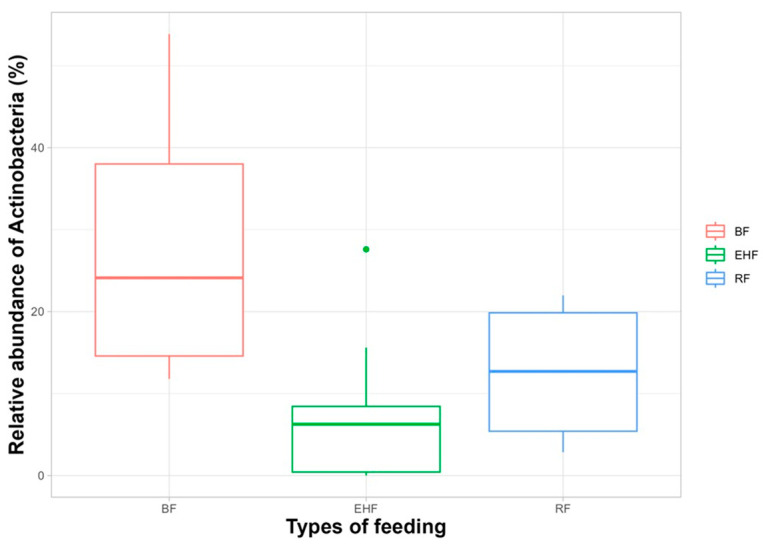
Relative abundance of the *Actinobacteria* phylum as a function of patient diet.

The analysis of fecal microbial 16S sequences showed differences in the composition of the gut microbial communities between the group of P-CMPA and the HC at 6 months of diagnosis. The percentage of *Actinobacteria* sequences was found to be significantly reduced in P-CMPA compared to in the HCs (*p* value < 0.001) (Supplementary Figure S1). Furthermore, significant differences in the proportion of *Firmicutes* between controls and patients (*p* value = 0.013) were observed. In patients, regarding the type of feeding, differences were found between breastfed and EHF-fed patients, showing 28.47% of Actinobacteria levels in BF versus 7.29% in EHF (Figure 3). At the family level, sequences belonging to the family *Bifidobacteriaceae* were also found to be statistically more abundant in fecal samples from the control group compared to samples from the patients (Supplementary Table S1). The differences were also evident when the type of feeding was taken into account, with a notable reduction in *Bifidobacteriaceae* levels in EHF-fed patients compared to breastfed patients.

**Figure 4:**
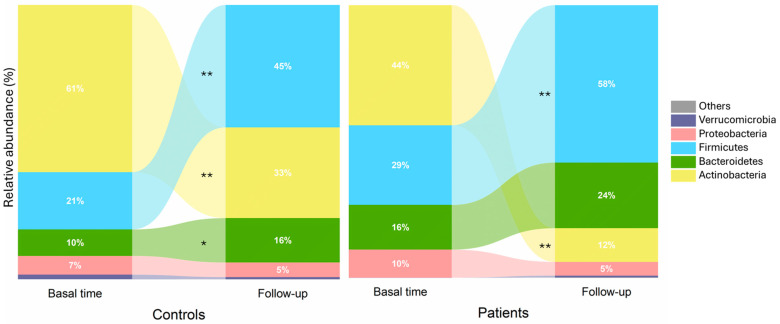
Evolutionary fecal microbiota profile between patients and controls at phylum level. Asterisks indicate significant statistical differences; *: *p* value ≤ 0.05, **: *p* value ≤ 0.01.

Significant changes were observed when comparing the microbial profiles at the time of diagnosis [15] with those of the same patients six months after the introduction of a milk-restricted diet (Figure 4). A decrease in Actinobacteria was observed in both controls and patients, although the reduction was more pronounced in the latter. Furthermore, an increase in the proportion of *Firmicutes* and *Bacteroidetes* was also observed (Figure 4).

### 3.4. Fecal Metabolic Concentration of Short-Chain Fatty Acids

Regarding the quantification of the main SCFAs in feces, no significant differences were observed in the concentration of acetic, butyric and propionic acids between patients and controls (Supplementary Table S2). With regard to branched-chain fatty acids (isobutyric and isovaleric acids), which are associated with the microbial catabolism of proteins, no significant variations were observed between any of the groups.

**Figure 5:**
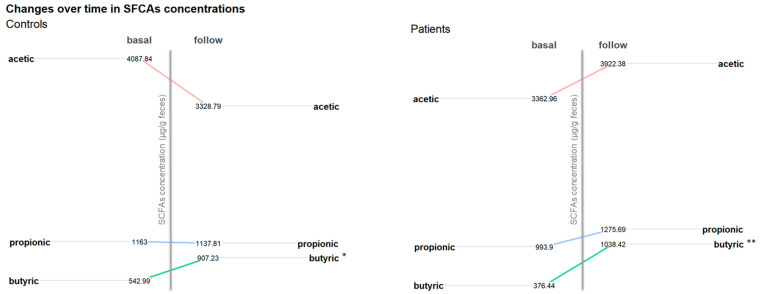
Changes in fecal short-chain fatty acids concentrations over time in controls and patients. Asterisks indicate significant statistical differences; *: *p* value ≤ 0.05, **: *p* value ≤ 0.01.

To assess the variation in fecal SCFAs over time, the data presented in Figure 5 were analyzed. A significant increase in the mean butyric acid levels was observed between the time of diagnosis and the six-month follow-up for both patients and controls (*p*-values = 0.006 and 0.017, respectively) (Figure 5).

## 4. Discussion

Food allergy is one of the most common chronic diseases of childhood, with an increasing prevalence in recent decades [24]. In this category, CMPA is a prevalent issue among infants and young children worldwide [25]. The age of diagnosis of CMPA in children is variable. It has been observed that in more than half of the subjects, the milk allergy resolves by a median age of 63 months [26]. We performed a prospective longitudinal study to compare anthropometric measurements, dietary patterns and the fecal microbial profile between infants with CMPA and healthy controls. In this cohort, the age of the patients at diagnosis was 4.46 ± 2.75 months. Follow-up was performed six months after the avoidance of dairy products in the diet. During this period, all patients remained allergic after tolerance testing. As mentioned above, some infants dropped out of the study. The mean ages of the controls and patients at the follow-up were 11.79 ± 0.57 and 12.24 ± 3.90 months, respectively.

Infants diagnosed with CMPA must avoid dairy products [27]. Among the possible treatment alternatives, continued breastfeeding is the preferred choice due to its advantageous effects for both the infant and the mother. Among the available alternatives, expert guidelines recommend the use of extensively hydrolyzed formulas or amino acid formulas for children with severe symptoms [28]. In addition, the use of vegetable-based formulas, mainly hydrolyzed rice and soy, are also appropriate options [29]. However, the latter formulas are consumed by a smaller percentage of children, indicating a gap in adherence to expert guidelines [30]. These data are consistent with the findings of our study population, in which it was observed that the highest percentage corresponds to patients with a diet based on hydrolyzed formulas (62% of patients), while only four patients used rice formulas.

The long-term management of CMPA includes monitoring the child’s growth and development. Jardim-Botelho et al. found no differences in growth status between controls and patients with CMPA at baseline. However, infants with CMPA on a cow milk-restricted diet had higher weight and length at 18-month follow-up [31]. Our results show no differences in growth between patients and controls at baseline or after 6 months of dietary restriction. In this regard, our findings are consistent with those of intervention studies in which patients with CMPA fed milk-replacement formulas showed adequate growth [27,32,33].

Nutritional considerations are important, as children with CMPA may be at risk for reduced intake of essential nutrients found in cow’s milk, including protein, fat, calcium and vitamins D and B12 [34]. It is of paramount importance to emphasize that in the event of a diagnosis of CMPA in children, one of the recommended courses of action is to continue breastfeeding as long as the mother is restricted in CMP. In this regard, the composition of human breast milk is complex and varies according to the needs of the child. In addition, the composition of breast milk changes dynamically over time, adapting to the changing needs of the infant. Some studies have observed differences in protein intake between patients fed hydrolyzed formula and healthy breastfed controls [35,36]. Other longitudinal studies have compared growth in patients with CMPA and concluded that there are no differences in growth depending on the type of feeding [37]. These data are consistent with the results of our study when comparing controls and patients. However, we observed a significant reduction in lipid intake, especially in SFA consumption, in patients compared to controls at follow-up, which deserves further investigation. Among CMPA patients, those who continued to breastfeed had a higher intake of lipid components than those who consumed EHF.

With regard to micronutrients, our results indicate disparities in vitamin intake, with the CMPA group exhibiting higher levels. Data on the micronutrient status are scarce in children on cow’s milk-exclusion diets. In contrast to the results of our own study, the research conducted by Kvammen and colleagues [38] showed that children with CMPA on a restricted diet exhibited deficiencies in vitamin D. However, the study did not have a healthy reference group. In a more recent study on six-month-old infants with CMPA, no significant differences in vitamin D or other micronutrient intakes were observed between breastfed and formula-fed infants [39]. Given the inconsistency of published data on nutrient intake and nutritional status in children on elimination diets, further research is needed to clarify the role of micronutrients and cow’s milk-exclusion diets [38].

The development of CMPA is complex and involves interactions between multiple factors, including genetic and environmental factors, as well as the gut microbiota [40]. Research has highlighted the relationship between the gut microbiota and food allergies, emphasizing the symbiotic role of the gut microbiota in immune development and gut integrity [14,41,42,43]. Changes in the composition of the gut microbiota have been observed in infants with CMPA, suggesting a potential association between the gut microbiota and the allergy [44]. Furthermore, a *Bacteroidetes*-enriched microbiota has been associated with proinflammatory responses in infants with CMPA [45]. These children have been found to exhibit increased diversity and altered microbial composition depleted of specific commensal bacteria, such as bifidobacteria, compared to non-allergic infants [15,42].

SCFAs produced by the gut microbiota have also been identified as key factors in the protection against food allergies [46]. While Goldberg et al. observed significantly higher levels of SCFAs in the non-allergic controls compared to the food allergy group [47], our previous studies on CMPA patients did not show any differences [15,48]. In this study, we observed a tendency for the concentration of fecal butyric acid to increase after 6 months. This is consistent with the increase observed for some microbial families, particularly members of the *Lachnospiraceae* and *Ruminococcaceae* families (Supplementary Table S1), which include well-known butyrate producers [49]. Butyrate has been identified as a key factor in the development of tolerance mechanisms in patients with CMPA. Experimental studies have demonstrated the tolerogenic effects of butyrate through epigenetic mechanisms [50]. Despite a significant increase in butyrate levels in CMPA patients following six months of treatment, none of them developed tolerance in our study.

Breastfeeding has been associated with the shaping of the gut microbiota, potentially preventing allergies [42]. In particular, HMOs present in breast milk support the colonization of beneficial gut bacteria such as bifidobacteria and contribute to immune development [51,52]. Prolonging breastfeeding for at least six months, and preferably for the entire first year of life, has been shown to be an effective method of preventing the onset of allergies [53].

The cross-sectional design and the relatively long follow-up of patient growth in our investigation can be interpreted as one of its strengths. In this study, data were gathered on the subjects at the time of birth, at the time of diagnosis and six months after the subjects began avoiding their intake of cow’s milk products. This approach allowed us to monitor the growth of the patients and to compare them with a cohort of healthy subjects, thereby extending our knowledge in this field. Consequently, data on gut microbiota sequencing at diagnosis and following dietary restriction provide insight into the link between diet and microbiota. To our knowledge, this is the first prospective longitudinal study to compare anthropometric measurements, dietary patterns and the fecal microbial profile between infants with CMPA and healthy children in a developed country.

It is important to acknowledge the limitations of our study. Firstly, the number of infants included in the final analysis may not be sufficient to draw firm conclusions. Conversely, it would have been beneficial to have biochemical determinations of micronutrients and vitamins of interest in order to correlate with the intake data collected.

In conclusion, although the growth of CMPA infants fed with therapeutic formula after six months of treatment is similar to CMPA breastfed infants, there are differences in microbiota composition and macronutrient intake that underline the importance of continued breastfeeding in CMPA cases.

## Figures and Tables

**Table 1:** General characteristics of participants in the study.

		Controls (*n* = 24)	Patients (*n* = 21)	*p* Value
Gender	Male	12 (50.00%)	13 (61.90%)	
	Female	12 (50.00%)	8 (38.10%)	0.423
Born at term		24 (100.00%)	20 (95.20%)	0.365
Type of birth	Vaginal	17 (70.80%)	18 (85.70%)	
	C-section	7 (29.20%)	3 (14.30%)	0.231
Weight at birth (kg)		3.30 ± 0.54	3.34 ± 0.57	0.665
Height at birth (cm)		49.90 ± 2.80	49.30 ± 1.90	0.204
First allergic reaction age (m)		-	4.46 ± 2.75	-
Weight at diagnosis (kg)		7.40 ± 0.89	6.62 ± 2.04	0.044
Height at diagnosis (cm)		66.10 ± 2.50	63.80 ± 7.50	0.094
CMPA type	M-CMPA	-	12 (57.10%)	
	NIM-CMPA	-	9 (42.90%)	
Special infant dietary replacement	EHF	-	13 (62.00%)	
	RF	-	4 (19.00%)	
	BF	7 (29.17%)	4 (19.00%)	
	Others	17 (70.83%)	-	
Age at follow-up (m)		11.79 ± 0.57	12.24 ± 3.90	0.973
Weight at follow-up (kg)		9.41 ± 0.89	9.58 ± 1.53	0.982
Height at follow-up (cm)		75.50 ± 2.60	74.80 ± 4.30	0.192
Weight-for-age at follow-up (z-score)		0.11 ± 0.74	0.14 ± 1.28	0.927
Height-for age at follow-up (z-score)		0.35 ± 1.09	−0.10 ± 1.16	0.585
BMI-for-age at follow-up (z-score)	Underweight (−5.99 to <−1)	2 (8.33%)	4 (19.00%)	
	Normal weight (−0.99 to 0.99)	19 (73.17%)	11 (52.40%)	
	Overweight risk (1 to 1.99)	3 (12.50%)	6 (28.6%)	

Results derived from U-Mann–Whitney-test are presented as estimated marginal mean ± standard deviation, and differences in categorical variables are examined using chi-squared analysis and presented as percentage (%). M-CMPA, IgE-mediated cow’s milk protein allergy. NIM-CMPA. non-IgE-mediated cow’s milk protein allergy. BMI, body mass index. BF, breastfeeding. EHF, extensively hydrolyzed formula. RF, rice formula. Others include commercial growth formulas and cow’s milk.

**Table 2:** Estimated dietary energy and macronutrient intake amongst healthy controls and allergic patients at 6 months’ follow-up.

	Controls (*n* = 24)	Patients (*n* = 21)
Total energy (kcal/day)	1083.46 ± 169.26 (987.79–1208.71)	1088.87 ± 210.65 (923.81–1265.64)
Protein (g/day)	38.70 ± 11.38 (31.17–45.80)	37.88 ± 10.80 (29.96–43.02)
Lípids (g/day)	39.65 ± 8.65 (34.08–44.40) *	35.78 ± 9.39 (29.30–38.12)
SFA (g/day)	14.65 ± 5.15 (11.29–18.16) *	10.92 ± 5.54 (7.77 ± 15.58)
16:0 (Palmitic)	4.60 ± 2.95 2.29–6.56)	3.69 ± 3.04 (1.03 ± 6.18)
18:0 (Estearic)	1.24 ± 0.83 (0.68–1.81)	0.95 ± 0.96 (0.22–1.33)
MUFA (g/day)	14.84 ± 5.21 (10.90–19.14)	12.41 ± 6.42 (8.85–17.12)
16:1 (Palmitoleic)	0.49 ± 0.38 (0.20–0.68)	0.41 ± 0.47 (0.11–0.45)
18:1 (Oleic)	11.30 ± 5.59 (6.95–16.80)	10.40 ± 6.31 (4.69–15.56)
PUFA (g/day)	4.48 ± 1.84 (3.06–5.59)	4.69 ± 2.10 (4.23–5.88)
18:2 (Linoleic)	3.27 ± 1.75 (1.76–4.42)	3.38 ± 1.62 (2.38–4.69)
18:3 (Linolenic)	0.41 ± 0.24 (0.20–0.57)	0.45 ± 0.24 (0.24–0.59)
Carbohydrates (g/day)	142.94 ± 29.04 (118.96–163.21)	153.83 ± 37.56 (129.46–187.63)
Total sugars	69.28 ± 15.86 (59.91–77.22)	57.12 ± 26.14 (36.97–76.10)
Dietary fiber (g/day)	9.63 ± 4.62 (6.81–12.83) *	12.89 ± 5.39 (9.15–16.05)

Results derived from U-Mann–Whitney-test are presented as estimated marginal mean ± standard deviation and the interquartile ranges (percentile 25 and percentile 75). SFA, saturated fatty acids. MUFA, monounsaturated fatty acids. PUFA, polyunsaturated fatty acids. Asterisks * indicate significant statistical differences (*p* value ≤ 0.05) in a certain line.

**Table 3:** Daily intake of micronutrients in healthy controls and allergic patients at 6 months’ follow-up.

	Controls (*n* = 24)	Patients (*n* = 21)
**Antioxidants**		
Vitamin C (mg/d)	110.33 ± 50.90 (68.85–158.95) *	145.62 ± 56.71 (102.91–187.02)
Vitamin E (mg/d)	6.39 ± 4.23 (3.26–8.78) *	9.04 ± 3.65 (6.65–11.87)
**B Vitamins**		
Vitamin B-1 (mg/d)	0.88 ± 0.46 (0.52–1.31)	0.91 ± 0.30 (0.66–1.14)
Vitamin B-2 (mg/d)	1.21 ± 0.62 (0.85–1.37)	1.09 ± 0.36 (0.84–1.29)
Vitamin B-3 (mg/d)	10.16 ± 4.1 (7.82–11.69) **	14.34 ± 4.58 (10.88–18.01)
Vitamin B-6 (mg/d)	1.40 ± 0.52 (1.02 ± 1.72)	1.49 ± 0.41 (1.07–1.73)
Folic acid (µg/d)	185.92 ± 83.57 (118.40–247.77)	218.11 ± 120.28 (130.04–259.84)
Vitamin B-12 (µg/d)	2.02 ± 1.03 (1.32–2.62)	1.83 ± 0.75 (1.41–2.28)
**Bone-related nutrients**		
Calcium (mg/d)	683.57 ± 276.18 (467.39–909.95)	562.02 ± 183.62 (430.83–654.23)
Phosphorus (mg/d)	623.14 ± 195.43 (494.72–732.93)	542.34 ± 183.62 (494.27–607.00)
Magnesium (mg/d)	144.92 ± 37.58 (118.72–175.72)	139.98 ± 43.99 (111.80–163.73)
Vitamin D (µg/d)	5.72 ± 4.06 (1.55–8.92) *	8.9. ± 5.38 (6.66–10.88)
**Other micronutrients**		
Sodium (mg/d)	467.70 ± 265.02 (280.88–550.44)	369.89 ± 163.48 (246.30–507.50)
Potassium (mg/d)	1880.49 ± 479.35 (1605.71–2126.69)	1799.43 ± 512.76 (1412.60–2211.90)
Iron (mg/d)	7.32 ± 3.42 (4.63–9.12)	9.78 ± 4.51 (6.13–11.29)
Selenium (µg/d)	35.84 ± 24.29 (24.42–41.98)	29.80–16.31 (16.84–38.39)
Manganese (µg/d)	2.71 ± 5.94 (0.74–1.56)	1.06–0.46 (0.71–1.37)
Iodine (mg/d)	67.26 ± 42.55 (36.77–94.28)	67.57 ± 27.95 (56.95–90.07)
Zinc (mg/d)	4.97 ± 2.39 (3.39–6.61)	5.12 ± 1.75 (4.14–6.30)
Vitamin A/ (µg/d)	741.72 ± 264.11 (584.58–912.13)	841.16 ± 426.23 (553.55–1119.80)
Vitamin K (µg/d)	49.57 ± 34.00 (27.13–60.29) *	125.42 ± 207.17 (44.48–90.09)

Results derived from U-Mann–Whitney test are presented as estimated marginal mean ± standard deviation and the interquartile ranges (percentile 25 and percentile 75). * and ** indicate significant statistical differences, *p* value ≤ 0.05 and *p* value ≤ 0.01, respectively.

## Data Availability

The raw sequence data were deposited in the Sequence Read Archive (SRA) of the NCBI (https://www.ncbi.nlm.nih.gov/sra) submitted on 20 June 2024 under BioProject ID PRJNA1126113 (sequence library IDs SAMN41921760—SAMN41921804) and released on 9 September 2024.

## Supplementary Materials

The following supporting information can be downloaded at https://www.mdpi.com/article/10.3390/children11091113/s1: Table S1: Differences in microbial relative abundance (%) at family level between patients with cow’s milk protein allergy and controls; Table S2: Fecal fatty acid concentrations comparing control and cow milk protein allergy patients; Figure S1: Relative abundance (%) of assigned sequences at phylum level in feces of patients and controls. * and ** indicate significant statistical differences, *p* value ≤ 0.05 and *p* value ≤ 0.01, respectively.
