# Supplementary material for: Evolutive Study of Dietary Aspects and Intestinal Microbiota of Pediatric Cohort with Cow’s Milk Protein Allergy
Source: Children (Basel). 2024 Sep 12;11(9):1113. doi: 10.3390/children11091113 (PMC11429558; doi:10.3390/children11091113)

Suppl. Table S1. Differences in microbial relative abundance (%) at family level between patients with cow's milk protein allergy and controls

	Controls (n=24)	Patients (n=21)
Bifidobacteriaceae	30.30 ± 21.28**	9.11 ± 12.15
Bacteroidaceae	13.07 ± 9.82	15.12 ± 13.98
Coriobacteriaceae	2.40 ± 2.77	1.48 ± 2.33
Prevotellaceae	0.75 ± 2.36	3.76 ± 8.46
Rikenellaceae	1.13 ± 1.54	0.87 ± 1.84
Enterococcaceae	0.26 ± 0.36	1.58 ± 5.46
Streptococcaceae	2.40 ± 2.93**	1.03 ± 1.89
Lachnospiraceae	24.39 ± 12.36	32.75 ± 15.63
Ruminococcaceae	12.83 ± 9.01*	18.91 ± 19.78
Erysipelotrichaceae	1.11 ± 1.08	1.04 ± 0.92
Veillonellaceae	2.04 ± 2.44	1.29 ± 1.80
Enterobacteriaceae	4.29 ± 7.33	7.63 ± 21.77

Data shown mean relative abundance ± standard deviation. U- Mann Whitney tests were used to evaluate differences in relative abundances among groups. * and ** indicate significant statistical differences; p value ≤0.05 and p value ≤0.01, respectively. Only families with a mean relative abundance higher than 1% in more than half of the samples for each group were presented.

Suppl. Table S2. Fecal fatty acids concentrations compared control and cow milk protein allergy patients

	Controls (n=24)	Patients (n=21)	p value
Acetic acid	3328.79 ± 1134.92	3922.38 ± 1591.79	0.187
Propionic acid	1137.81 ± 466.71	1275.69 ± 756.63	0.682
Butyric acid	907.23 ± 601.50	1038.42 ± 881.79	0.820
Isobutyric acid	135.41 ± 97.23	107.32 ± 84.27	0.334
Isovaleric acid	225.28 ± 170.71	176.65 ± 155.20	0.187
Valeric acid	7.35 ± 8.39	8.38 ± 11.15	0.835
Caproic acid	6.23 ± 14.55	42.51 ± 83.23	0.574
Acetic/propionic	3.37 ± 1.77	4.13 ± 3.53	0.785

Data shown mean relative abundance ± standard deviation. U- Mann Whitney tests were used to evaluate differences in relative abundances among groups.

Suppl. Figure S1. Relative abundance (%) of assigned sequences at phylum level in feces of patients and controls. * and ** indicate significant statistical differences; p value ≤0.05 and p value ≤0.01, respectively.

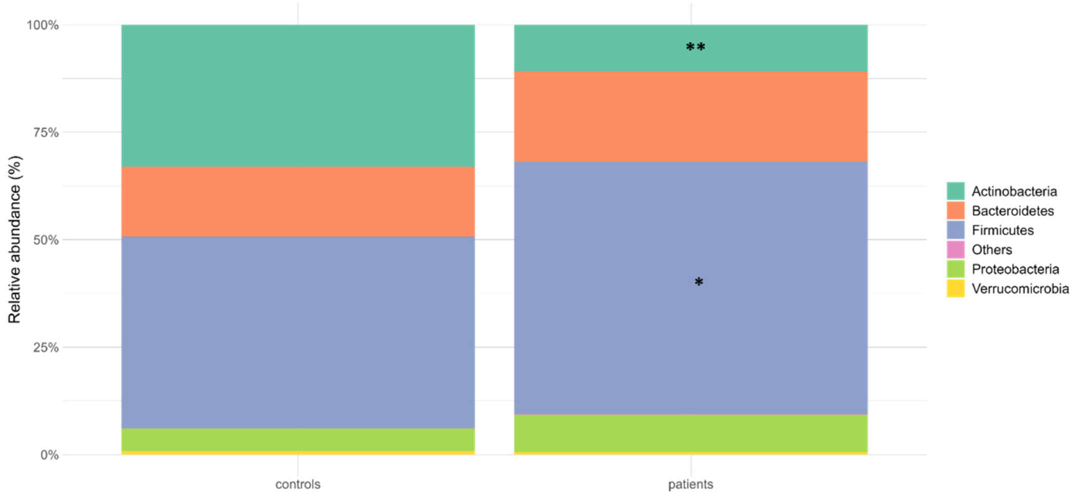

Supplement: Supplementary file 1 [file children-11-01113-s001.zip › children-3176760-supplementary.pdf]
